# Contribution of NKT cells and CD1d-expressing cells in obesity-associated adipose tissue inflammation

**DOI:** 10.3389/fimmu.2024.1365843

**Published:** 2024-02-15

**Authors:** Masashi Satoh, Kazuya Iwabuchi

**Affiliations:** Department of Immunology, Kitasato University School of Medicine, Sagamihara, Japan

**Keywords:** NKT cells, CD1d, macrophages, obesity, insulin resistance, adipose tissue inflammation

## Abstract

Natural killer T (NKT) cell are members of the innate-like T lymphocytes and recognizes lipid antigens presented by CD1d-expressing cells. Obesity-associated inflammation in adipose tissue (AT) leads to metabolic dysfunction, including insulin resistance. When cellular communication is properly regulated among AT-residing immune cells and adipocytes during inflammation, a favorable balance of Th1 and Th2 immune responses is achieved. NKT cells play crucial roles in AT inflammation, influencing the development of diet-induced obesity and insulin resistance. NKT cells interact with CD1d-expressing cells in AT, such as adipocytes, macrophages, and dendritic cells, shaping pro-inflammatory or anti-inflammatory microenvironments with distinct characteristics depending on the antigen-presenting cells. Additionally, CD1d may be involved in the inflammatory process independently of NKT cells. In this mini-review, we provide a brief overview of the current understanding of the interaction between immune cells, focusing on NKT cells and CD1d signaling, which control AT inflammation both in the presence and absence of NKT cells. We aim to enhance our understanding of the mechanisms of obesity-associated diseases.

## Introduction

Inflammation/immune response control obesity in the adipose tissue (AT) of the body (1, 2). Obesity is a low-grade chronic inflammatory disease that contributes to metabolic dysfunction and insulin resistance (3, 4). Initially, hypertrophied adipocytes secrete inflammatory cytokines and chemokines, thereby recruiting immune cells that promote AT inflammation, including macrophages (Mϕs), T cells, B cells, and neutrophils (5–11). While various immune cells contribute to AT homeostasis and inflammation, natural killer T (NKT) cells play a crucial role in developing obesity and insulin resistance (12). NKT cells are a unique subset of T cells that recognize lipid antigens on MHC class I-like CD1d molecules (13–15). NKT cells are further classified into two subsets depending on their T cell receptor (TCR) expression: type I NKT (invariant NKT; iNKT) cells that harbor an invariant TCR α-chain (Vα14-Jα18 in mice and Vα24-Jα18 in humans) and type II NKT (variant NKT; vNKT) cells that express diverse TCRs. iNKT cells recognize a prototypical ligand, α-galactosylceramide (α-GalCer), whereas vNKT cells recognize various lipid antigens, including sulfatide (16–18). NKT cells secrete various cytokines/chemokines/cytocidal molecules, activating and recruiting immune cells to eliminate target cells (19). Furthermore, iNKT cells can be categorized based on the expression of transcription factors such as T-bet for NKT1 cells, GATA3 and PLZF for NKT2 cells, and RORγt for NKT17 cells (20, 21), which may contribute to the development of inflammatory diseases, including obesity (22–24).

The contribution of CD1d itself to cellular signaling, aside from its role in ligand presentation to NKT cells in inflammatory and immune responses, should be considered. Recent reports have demonstrated that CD1d can modulate critical responses through its cytoplasmic portion (25–30). In this review, we discuss how NKT cells contribute to inflammation through interaction with CD1d-expressing cells in AT and how the intrinsic function of CD1d itself may influence the response in an NKT cell-independent manner.

## Roles of NKT cells in diet-induced obesity using global CD1d knockout mice

Numerous studies have revealed that AT-resident and infiltrating immune cells control obesity-associated AT inflammation in a DIO model by feeding mice a high-fat diet (HFD; 60% fat kcal). T helper type 1 (Th1)-immune responses formed by M1-Mϕs, CD8^+^ T cells, NK cells, and ILC1s exacerbate AT inflammation and insulin resistance (5, 7, 31–34). In contrast, Th2-immune responses induced by M2-Mϕs, regulatory T cells, eosinophils, and ILC2s improve insulin sensitivity (8, 35, 36). Additionally, the ILC2s-eosinophils-M2-Mϕs circuit plays a central role in differentiating beige fat from white fat (beiging), where beige fat expresses thermogenic genes to defend against cold and obesity (37–39). Thus, in the steady state, a lean Th2-immune environment is maintained by the AT-resident cells.

NKT cells appear to play dual roles in the development of obesity. Reports have shown that both iNKT and vNKT cells promote AT inflammation and insulin resistance (40–43), whereas other studies have indicated that iNKT cells play either protective or neutral roles against obesity in DIO experiments using CD1d KO and Jα18 KO mice (44–49) (**Table 1**). Jα18 KO mice exhibit a significant reduction in T-cell receptor (TCR) diversity (52), thus mucosal-associated invariant T (MAIT) cells which utilize Jα33 are lost (53). Considering that MAIT cells also impact the development of obesity (54–57), Traj18 KO mice (distinct from Jα18 KO mice here) generated by depleting only the Traj18 locus, essentially represent iNKT cell KO mice (42). AT-resident iNKT cells express E4BP4 but not PLZF, reflecting their anti-inflammatory phenotype, and IL-10-producing NKT cells (NKT10) are enriched in subcutaneous white AT (58, 59). Moreover, when the F108Y substitution was artificially induced in TCRβ (Vβ8.2), thereby reducing the interaction of mutated iTCR with CD1d by a partial disruption of the hydrophobic patch formation with TCR α and β chain pairing, it altered iNKT cell development to an E4BP4-expressing, AT-resident-like phenotype (60). Thus, AT-resident iNKT cells may differentiate and exhibit unique functions compared to iNKT cells located in other tissues (61).

**Table 1:** Role of NKT cells in a murine DIO model.

Strain	KO/Tg mice, NKT cell agonist	Diet (fat source)	Compared to control		NKT cell responses in DIO	Ref
			Body weight	Insulin resistance		
C57BL/6	CD1d KO	HFD (soybean oil, lard)	↓	improved		(41)
C57BL/6	Jα18 KO	HFD (soybean oil, lard)	↓	improved	
C57BL/6	αGC administration	HFD (soybean oil, lard)	→	worsened	produce TNF-α, IFN-γ
C57BL/6	CD1d KO	HFD (safflower oil, beef tallow)	↓	improved		(40)
C57BL/6	Jα18 KO	HFD (safflower oil, beef tallow)	→	not changed	
C57BL/6	αGC administration	HFD (safflower oil, beef tallow)	→	worsened	
C57BL/6	CD1d KO	HFD (soybean oil, lard)	↓	no data		(42)
C57BL/6	Traj18 KO	HFD (soybean oil, lard)	↓	improved	
C57BL/6	Vα14Tg/Ldlr KO	high fat, high sucrose, 0.15% cholesterol	↑	worsened		(43)
Balb/c	CD1d KO	HFD (soybean oil, lard)	→	not changed		(48)
C57BL/6	CD1d KO	HFD (soybean oil, lard)	→	worsened		(49)
C57BL/6	Jα18 KO	HFD (soybean oil, lard)	→	not changed	
C57BL/6	CD1d KO	HFD (soybean oil, lard)	↑	worsened	produce IL-10	(44)
C57BL/6	Jα18 KO	HFD (soybean oil, lard)	↑	worsened	
C57BL/6	αGC administration	HFD (soybean oil, lard)	↓	improved	
C57BL/6	CD1d KO	HFD (soybean oil, lard)	no data	not changed		(45)
C57BL/6	αGC administration	HFD (soybean oil, lard)	no data	improved	IL-4/STAT6 dependent
C57BL/6	CD1d KO	both LFD and HFD (soybean oil, lard)	→	worsened		(46)
C57BL/6	Jα18 KO	LFD	no data	worsened	
C57BL/6	αGC administration	LFD	no data	not changed	produce IL-4, IL-13
C57BL/6	αGC administration	HFD (soybean oil, lard)	↓	improved	produce IL-13	(47)
C57BL/6	sulfatide	HFD (soybean oil, lard)	↓	improved
C57BL/6	Adipoq-cre-Cd1d1^f/f^	HFD (safflower oil, beef tallow)	↓	improved	produce IFN-γ	(50)
C57BL/6	Adipoq-cre-Cd1d1^f/f^	HFD (soybean oil, lard)	→	worsened	produce IL-4	(51)
C57BL/6	LysM-cre-Cd1d1^f/f^	HFD (safflower oil, beef tallow)	→	worsened	produce IFN-γ	(24)
C57BL/6	CD11c-cre-Cd1d1^f/f^	HFD (safflower oil, beef tallow)	→	improved		(24)

NKT cell-deficient mice do not show any pathogenic phenotype in comparison with WT mice, either in an obese or lean state under normal dietary conditions. However, when they are fed an HFD, NKT cells function as either pro-obese or pro-lean (40, 41, 44, 45, 49). This implies that NKT cells in HFD-fed mice are activated by unknown endogenous ligands presented by CD1d^+^ APCs, including flora-derived ligands (62–64), presumably even during obesity. Schipper et al. showed that CD1d KO mice exhibited adipocyte dysfunction and insulin resistance even under steady-state conditions (46), suggesting that NKT cells function in both obesity and a lean state. Alternatively, the dysfunction may be primarily attributed to the deficiency of CD1d molecules in adipocytes, as discussed later. Moreover, the phenotype of CD1d KO and Jα18 KO mice fed an HFD varied among laboratories (12), ranging from lean to obese, including a neutral state, compared to WT mice. Although contradictory results have been reported, presumably due to differences in the lipid composition of the HFD and intestinal microbiota for different murine strains, the critical reason for the discrepancy remains elusive. Selvanantham et al. have reported that CD1d KO mice exhibit altered gut microbiome profiles, characterized by increase in segmented filamentous bacteria and decrease of *Akkermansia*, which exacerbate intestinal inflammation (65). *Bacteroides fragilis* produces the glycosphingolipid α-GalCer_Bf_, which is structurally related to the prototypic ligand α-GalCer or KRN7000 (62), exhibiting regulatory activity by iNKT cells. This indicates that some bacteria in the intestine are involved in the functional modulation of NKT cells, however, whether it indeed affects the function of NKT cells during obesity has not been examined. Meanwhile, the composition and fatty acid concentration in sera, altered and elevated in obese subjects, are determined based on endogenous synthesis rates and dietary fat characteristics (66, 67). Although the ligand for NKT cells in adipocytes remains elusive, it appears possible that dietary-derived lipid components are involved in their ligand production. At least, alteration in serum fatty acid composition affects Mϕs, via TLR4 and other receptors, which may modulate NKT cell activation in an indirect fashion (24). Additionally, single-cell analysis have unveiled distinct subsets of AT-resident iNKT cells, including AT-iNKT10 (induction of Tregs and M2-Mϕs), AT-iNKT1 (killing pathogenic Mϕs via NK cells and clearance of dead adipocytes via Mϕs) and AT-iNKT17 (via induction of adipose stem cell proliferation by amphiregulin) (68, 69). Of significance is the inquiry into how AT-iNKT cells are generated and activated, representing a likely major contribution of the adipose tissue microenvironment. Additionally, from the perspective of the CD1d molecule itself, the activation of the CD1d is differentially regulated by endogenous/exogenous lipid ligands which are biosynthesized during obesity, or through the modulation of the CD1d signaling cascade, as indicated by reports suggesting that endogenous ligands switch on CD1d activation (28, 29). Hence, further investigation is necessary to reconcile this contradiction and establish a therapeutic strategy for obesity-associated diseases.

## NKT cell-adipocyte interactions in AT using tissue-specific CD1d KO mice

NKT cells are activated in AT by interacting with CD1d-expressing cells, including Mϕs, dendritic cells, adipocytes, and leukocytes, such as eosinophils. AT functions as an endocrine organ, as adipocytes secrete adipokines and store triglycerides for energy (70). Several studies have demonstrated that adipocytes activate both T and NKT cells through antigen presentation (50, 51, 71–73). CD1d expressed on the surface of adipocytes can induce iNKT cell activation depending on the expression of microsomal triglyceride transfer protein (MTP) and CCAAT/enhancer-binding protein (C/EBP)-β and -δ, even in the absence of exogenous ligands, suggesting that adipocytes express endogenous ligands recognized by NKT cells (71). The inhibition of UDP-glucose ceramide glucosyltransferase (UGCG), the first rate-limiting step in the glucosylceramide biosynthesis pathway, resulted in decreased iNKT cell activity (74). This finding suggests that the amount of glucosylceramide influences the biosynthetic pathway of lipid self-antigen presentation by adipocytes in iNKT cells. Additionally, during their interaction with adipocytes within a lipid-rich microenvironment, the cytokine output of NKT cells skews towards IFN-γ rather than IL-4 (75), indicating that lipid conditions, such as the balance of fatty acids, are important factors for NKT cell activation by adipocytes. Furthermore, the LDL-α-GalCer complex elicits a stronger iNKT cell response than α-GalCer alone; while, LDL receptor mutation impairs their activation. Thus, lipoproteins can form complexes with lipid antigens to facilitate LDL receptor-mediated uptake by APCs, leading to enhanced iNKT cell activation (76).

Our group has demonstrated the role of NKT cell-adipocyte interactions *in vivo* using adipocyte-specific *Cd1d1*-deficient (AdipoqCre-*Cd1d1* ^f/f^) mice during obesity. In comparison to control mice fed a HFD, AdipoqCre-Cd1d1^f/f^ mice exhibited suppressed body weight gain and insulin resistance, suggesting that the interaction between iNKT cells and adipocytes plays a pro-inflammatory role in AT. iNKT cells activated by adipocytes secrete IFN-γ, which enhances the expression of CD1d and CCL2 in adipocytes, thereby promoting a positive loop for AT inflammation (50).

In contrast, other reports have shown that a similar conditional knockout mouse, adipocyte-specific CD1d-KO (CD1d^ADKO^), exhibited reduced IL-4 expression in adipose iNKT cells, resulting in aggravated AT inflammation and insulin resistance in HFD-fed mice. This implies that iNKT cells stimulated by CD1d-expressing adipocytes induce anti-inflammatory responses in the AT (51). Furthermore, the results observed in CD1d^ADKO^ mice recapitulated those in whole-body CD1d KO mice, demonstrating the opposite conclusion.

Recently, Xiao et al. re-expressed CD1d by transferring the *Cd1d* gene into the visceral AT (VAT) of CD1d KO mice using an adeno-associated viral (AAV) vector to investigate the interactions between adipocytes and immune cells. The mice with the *Cd1d* gene transferred showed massive expansion of CD8^+^ T cells in the VAT, leading to the dysregulation of adipocyte functions through the activation of the NLRP3 inflammasome (77). Although the transduced mice exhibited a 25% reduction in VAT weight, there was no significant change in other parts of the AT. Since the mice were NKT cell-deficient and *CD1d*-gene in AAV appeared not to be expressed in either CD4^+^CD8^+^ thymocytes or thymic epithelial cells, CD1d expressed on adipocytes served as a neoantigen introduced like an allograft, inducing responses by CD8^+^ T cells along with an increase in CD4^+^ and CD4^-^8^-^ T cells. It would be of an interest whether iNKT cells or vNKT cells respond in CD1d-AAV transduced WT or Jα18 KO mice fed on HFD.

## NKT cell-macrophage interactions in AT using cell lineage-specific CD1d KO mice

Mϕs are abundantly present in AT and play a crucial role in maintaining AT homeostasis. Mϕs are phenotypically and functionally classified into two types: pro-inflammatory M1 and anti-inflammatory M2, based on gene expression and markers. Adipose iNKT cells have been shown to interact with Mϕ, which act as antigen-presenting cells that polarize Mϕs towards M2 under the influence of IL-10 (44). To examine the role of the interaction between NKT cells and Mϕs during obesity, we utilized myeloid-specific Cd1d1-deficient (LysMCre-*Cd1d1* ^f/f^) mice. If the interaction is beneficial, its disruption between iNKT cells and Mϕ should result in AT inflammation and obesity. However, LysMCre-*Cd1d1* ^f/f^ mice gained body and VAT weights similar to those of control mice and exhibited enhanced insulin resistance, which was associated with M1-Mϕ and a bias toward NKT1/Th1 in the AT. The insulin resistance result aligns with the findings of a previous study (44), while the weight gain was not significantly greater than that of the control mice. These results may be primarily interpreted as defective M2 polarization due to the lack of interaction between iNKT cells and Mϕ via CD1d. Additionally, CD1d-deficient Mϕs expressed more IL-12p40 than control Mϕs in response to palmitic acid, and the inflammatory phenotype was enhanced by direct contact with iNKT cells (24).

Alternatively, these results may indicate that CD1d-deficient Mϕs themselves exhibit an inflammatory phenotype upon TLR stimulation (palmitic acid as a TLR4 ligand), and the interaction with iNKT cells strengthens the TLR responses, however, the mechanism of interaction remains elusive. A previous report by Zhang et al. suggested that the iNKT cell-Mϕ interactions are important in controlling AT inflammation during obesity. They employed LysMCre-*Cd1d1* ^f/f^ mice as M2-specific CD1d-deleted mice, induced inflammation and insulin resistance during obesity due to the inhibition of M2-Mϕ and iNKT cell interactions (78). M2-Mϕ express more CD1d than M1-Mϕ in murine VAT, facilitating the induction of IL-4 and IL-13 by interacting with iNKT cells (44, 78).

In humans with diabetes, CD11c^+^ VAT Mϕs laden with several lipids express more CD1d than CD206^+^ VAT Mϕs, although functional profiling such as M1 or M2 classification is not clear based on surface markers, thus implying that the signatures of human ATMϕ subtypes are unique (79, 80). In the presentation of antigens via CD1d, Th1-biasing glycolipids such as α-GC C26:0 (both Th1+Th2) and α-C-GC C26:0, which stimulate much lower IL-4 and relatively higher and more prolonged IFN-γ secretion, have consistently been observed to form complexes with CD1d that preferentially localize to cholesterol-rich membrane rafts. Contrarily, CD1d with Th2-biasing glycolipids such as α-GC C20:2, α-GC C20:1, α-GC C18:3, and α-GC C10:0, which stimulate strong IL-4 secretion relative to IFN-γ, are more evenly distributed throughout the cell membrane. Neutralization of lysosomal pH enhanced the localization of the CD1d-Th2-biasing glycolipid complex to lipid rafts on the plasma membrane, although the presentation of Th1-biasing glycolipids was drastically reduced. These results suggest that lysosomal pH controls the stability and localization of CD1d-glycolipid complexes in lipid rafts by modulating the cytokine output of iNKT cells (81, 82). Lysosomes accumulate in lipid-laden ATMϕs and appear to be important for lipid metabolism (83). However, it is unclear whether lysosomal activation and pH are involved in M1/M2 Mϕ polarization and obesity development.

In addition to Mϕs, DCs contribute to the control of AT homeostasis and insulin sensitivity. Flt3 KO mice lacking DCs or CD11c^+^ cell-depleted mice treated with diphtheria toxin failed to induce obesity, insulin resistance, and liver steatosis compared to that in control mice during HFD feeding (84, 85). In contrast, Batf3 KO mice lacking cDC1 showed increased body weight and adiposity during aging, partially mediated by the cDC-iNKT cell axis. However, these data did not show the actual interaction between cDC and iNKT cells (86). In our model, inhibition of the interaction between iNKT cells and DC in CD11cCre-*Cd1d1* ^f/f^ mice demonstrated that insulin sensitivity was improved in obese mice compared to that in control mice (24). iNKT cells activated by adipose DCs produced more IFN-γ than those activated by adipose Mϕs, and different phenotypes were observed between LysMCre-*Cd1d1* ^f/f^ and CD11cCre-*Cd1d1* ^f/f^ mice. These findings suggest that NKT cells play different roles in the development of obesity by interacting with adipose Mϕs or DCs. However, it is unclear whether the lack of CD1d affects the functions of CD1d-expressing cells.

## The function of CD1d signaling in CD1d-expressing cells

Although the NKT cell-Mϕ interaction seems beneficial in obesity (24), CD1d-deficient Mϕs may exhibit pro-inflammatory functions independent of their lack of cellular interaction with iNKT cells. Given our results that BMMϕs derived from LysMCre-*Cd1d1* ^f/f^ mice express more IL-12p40 in a cell-autonomous manner, it is the deficiency (or downregulation) of CD1d molecules on the cell surface that causes Mϕs to produce more IL-12p40 in response to TLR4 stimulation. The cytoplasmic domain of CD1d transduces signals for the trafficking and regulation of inflammatory responses. The CD1d molecule contains a tyrosine-based signal (YXXZ) (where Y represents tyrosine, X represents any amino acid, and Z is a hydrophobic amino acid) that mediates intracellular trafficking, antigen presentation, NKT cell development (87–90), and a leucine-based basolateral sorting signal in the cytoplasmic tail (91). Another threonine residue (T322) and a serine residue (S323) in the cytoplasmic tail of CD1d control its transport to the cell surface and lysosomal degradation; thus, these motifs can regulate the functional expression of CD1d (92, 93). A few components for sorting CD1d into lysosomes, such as the AP-3 adaptor protein complex and MTP, are necessary to functionally express CD1d (94, 95).

Moreover, phosphorylation of tyrosine residues in the cytoplasmic tail is induced by CD1d crosslinking with an anti-CD1d antibody, which leads to upregulated expression of IL-10 in intestinal epithelial cells (25, 26). Conversely, CD1d crosslinking induces IL-12 production in monocytes and DC via NF-κB activation (27), indicating that the output of the CD1d crosslinking may vary depending on the cell type where CD1d is expressed. Similarly, CD1d in endosomal compartments binds isoglobotrihexosylceramide (iGb_3_), an endogenous ligand for iNKT cells, induces Tyr^332^ phosphorylation of the CD1d cytoplasmic domain, and synergizes with TLR signaling in Mϕs and DC (28). Cui et al. have reported that CD1d stimulated with iGb_3_ induces Ser^330^ dephosphorylation of CD1d cytoplasmic residue, followed by the downregulation NF-κB activation through the reduction of Peroxiredoxin 1 (PRDX1)-associated AKT-STAT1 phosphorylation in Mϕ (29). The short cytoplasmic tail of CD1d might mediate inhibitory signals for NF-κB activation, depending on Ser^330^ rather than Tyr^332^ residue. A similar signaling pathway has been studied as reverse signaling by MHC class Ia in immune and non-immune cells (96).

Notably, a recent study has shown that CD1d-deficient Mϕs amplify TLR signaling by increasing lipid uptake via CD36, independent of CD1d cytoplasmic signaling. This is demonstrated by the fact that the expression of CD1d with a deficient cytoplasmic tail (7 residues) or the tail mutant (^332^Y -> A) showed similar activity as that of the WT CD1d molecule (30).

According to above reports, the inhibitory effect of CD1d on TLR signaling is weakened in the Mϕs of LysMCre-Cd1d1^f/f^ mice, thereby potentiating the IL-12-IFN-γ circuit leading to AT inflammation (24). However, whether the signaling via CD1d influences only TLR responses or other factors, such as C-type lectins and scavenger receptors, remains unclear. These data suggest that it is difficult to distinguish whether the phenotype observed in CD1d-deficient mice is due to the lack of NKT cells or the CD1d deletion in the respective studies. Caution should be exercised when interpreting the results of studies using mice with either whole-body or conditional deletions of CD1d.

## Conclusion

**Figure 1:**
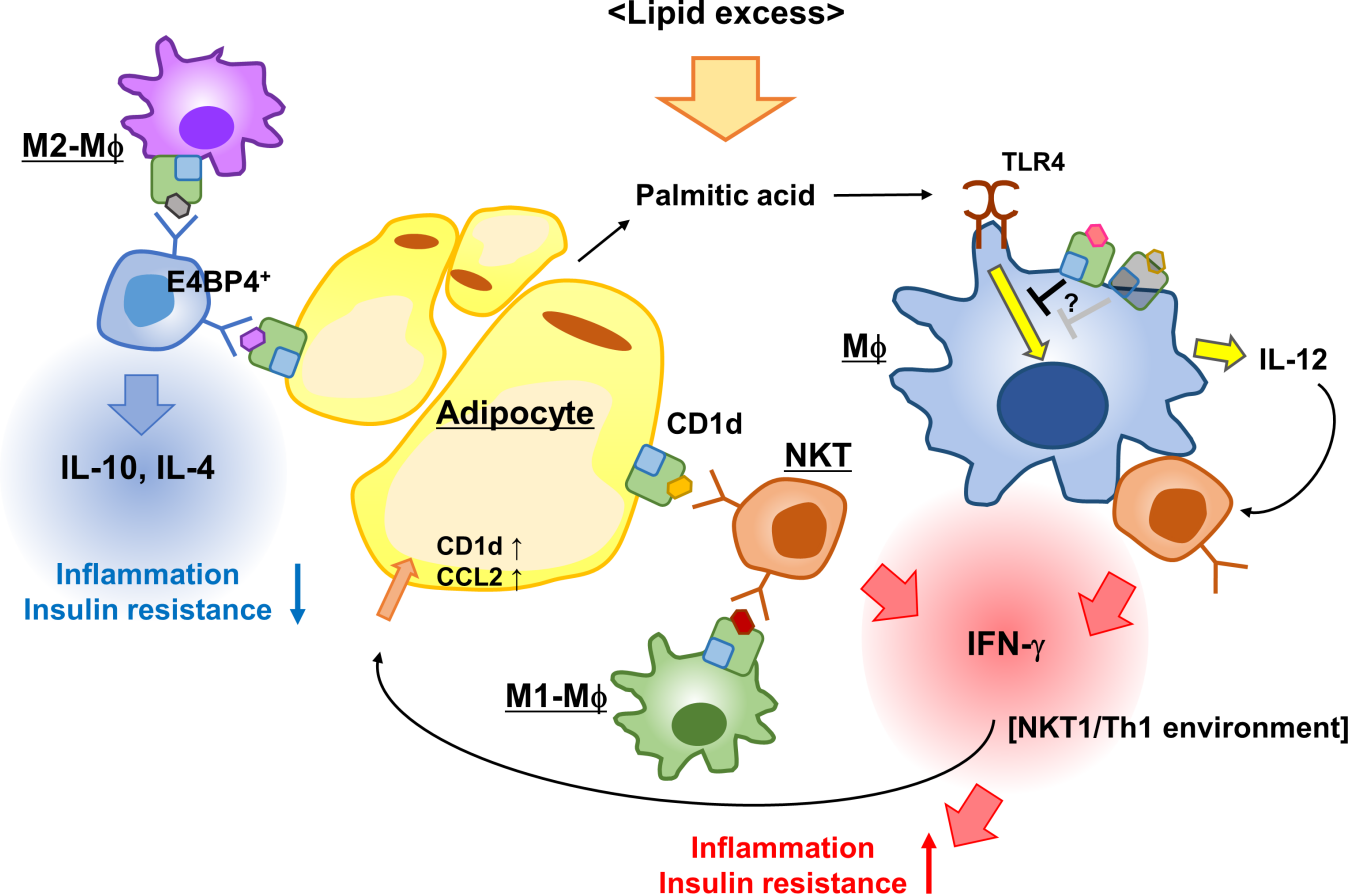
iNKT cells and CD1d control AT inflammation. Adipose iNKT cells interact with adipocytes, predominantly secreting IFN-γ and activating adipocytes to enhance the expression of CD1d and CCL2. The iNKT-adipocyte interaction forms an inflammatory circuit leading to AT inflammation and insulin resistance. The interaction between M2-Mϕ and iNKT cells induces Th2 cytokines, while the interaction with M1-Mϕ leads to Th1 cytokines, thereby modulating AT microenvironment. CD1d-deficient (or reduced) Mϕs secreted more IL-12 in response to palmitic acid than WT Mϕs to induce IFN-γ production in iNKT cells, because CD1d molecules presumably suppressed TLR responses in WT Mϕs. This scheme indicates that the activation process of adipose iNKT cells is APC-specific and that CD1d molecules contribute to immune responses.

NKT cells control obesity-associated AT inflammation by interacting with CD1d-expressing cells such as adipocytes, Mϕs, and DCs. In the interactions among these cells, unknown endogenous lipid ligands are presented via CD1d. Different ligands are possibly expressed depending on the cell type, leading to the respective immune microenvironment and phenotypes in the development of obesity (**Figure 1**). Additionally, NKT cells may contribute to both lean and obese phase, and their interaction with the predominant APCs at the time, such as M2-Mϕs in steady state AT or hypertrophied adipocytes in obesity, can affect NKT cell function. However, if CD1d molecules had a regulatory function that either enhances or suppresses the inflammatory process in Mϕs, this pathway could function independently of NKT cells. To understand the chronic inflammatory process during obesity, it will be important to further investigate the regulatory effect of CD1d, endogenous glycolipids, and NKT cells on immune responses in the AT. Thus, we obtained new insights into the strategies for overcoming obesity and obesity-associated diseases.

## Author contributions

MS: Writing – original draft, Writing – review & editing. KI: Writing – original draft, Writing – review & editing.
